# Chemical Constituents and Hypoglycemic Mechanisms of *Dendrobium nobile* in Treatment of Type 2 Diabetic Rats by UPLC-ESI-Q-Orbitrap, Network Pharmacology and In Vivo Experimental Verification

**DOI:** 10.3390/molecules28062683

**Published:** 2023-03-16

**Authors:** Zhaoyang Li, Meiling Zeng, Keyong Geng, Donna Lai, Zhi Xu, Wei Zhou

**Affiliations:** 1 School of Pharmacy, Guizhou Medical University, Guiyang 550025, China; 2 School of Medicine, Western Sydney University, Penrith, NSW 2751, Australia; 3 Guizhou Miaoaitang Health Management Co., Ltd., Guiyang 550025, China

**Keywords:** *Dendrobium nobile*, diabetes mellitus, hypoglycemic mechanism, UPLC-ESI-Q-Orbitrap

## Abstract

This study aimed to systematically explore the chemical constituents of *D. nobile* and its hypoglycemic effect by UPLC-ESI-Q-Orbitrap, network pharmacology and in vivo experiment. The chemical constituents of *D. nobile* were qualitatively analyzed, and the hypoglycemic compounds were quickly identified. Network pharmacological analysis and molecular docking technique were applied to assist in the elucidation of the hypoglycemic mechanisms of *D. nobile*. A type 2 diabetic mellitus (T2DM) rat model was established using the HFD and STZ method for in vivo experimental verification, and these T2DM rats were treated with *D. nobile* extract and *D. nobile* polysaccharide for two months by gavage. The results showed that a total of 39 chemical constituents of *D. nobile*, including alkaloids, bibenzyls, phenanthrenes and other types of compounds, were identified. *D. nobile* extract and *D. nobile* polysaccharide could significantly ameliorate the body weight, hyperglycemia, insulin resistance, dyslipidemia and morphological impairment of the liver and pancreas in the T2DM rats. α-Linolenic acid, dihydroconiferyl dihydro-p-coumarate, naringenin, *trans-N*-feruloyltyramine, gigantol, moscatilin, 4-*O*-methylpinosylvic acid, venlafaxine, nordendrobin and tristin were regarded as the key hypoglycemic compounds of *D. nobile*, along with the hypoglycemic effect on the PI3K-AKT signaling pathway, the insulin signaling pathway, the FOXO signaling pathway, the improvement of insulin resistance and the AGE-RAGE signaling pathway. The Western blotting experiment results confirmed that *D. nobile* activated the PI3K/AKT pathway and insulin signaling pathway, promoted glycogen synthesis via regulating the expression of glycogen synthase kinase 3 beta (GSK-3β) and glucose transporter 4 (GLUT4), and inhibited liver gluconeogenesis by regulating the expression of phosphoenolpyruvate carboxykinase (PEPCK) and glucose 6 phosphatase (G6pase) in the liver. The results suggested that the hypoglycemic mechanism of *D. nobile* might be associated with liver glycogen synthesis and gluconeogenesis, contributing to improving insulin resistance and abnormal glucose metabolism in the T2DM rats.

## 1. Introduction

Diabetes mellitus (DM), one of the most common chronic diseases, has become a significant threat to human health and an increasing global health burden [1]. More than 90% of the diabetic population are diagnosed with type 2 diabetes mellitus (T2DM), which is a disease characterized by chronic hyperglycemia, hyperlipidemia and insulin resistance, with disturbances of glucose, fat and protein metabolic disorders, which are all caused by insufficient insulin secretion and/or insulin resistance [2]. *Dendrobium nobile* (*D. nobile*) is a famous Chinese materia medica and common food ingredient that has been consumed for thousands of years in Chinese history. Many Chinese medical prescriptions, which have been clinically proven to be effective, contain *D. nobile*, including Shihuyin, Shihusan, and Shihu Yeguang pills, all of which are used in the treatment of clinical DM and its complications. Our previous study also confirmed that *D. nobile* had a good hypoglycemic effect, and that *D. nobile* alkaloids and *D. nobile* polysaccharides were effective for the treatment of DM and its complications, leading to an improvement in blood glucose level, kidney damage and liver steatosis in diabetic rats [3,4]. *D. nobile* phenols may also have hypoglycemic activity. However, the chemical constituents and hypoglycemic mechanisms of *D. nobile* are still unclear.

Because of its high-precision resolution, very low *m*/*z* error and high detection sensitivity [5], ultra high-performance liquid chromatography coupled with electron spray ionization and quadrupole-orbitrap mass spectrometry (UPLC-ESI-Q-Orbitrap) as a high-resolution mass spectrometry (HRMS) has been widely used to reveal the effectiveness of traditional Chinese medicines (TCMs) and unknown compounds. Natural product databases, including the SIOC chemical database and Reaxy database, have also been used to support UPLC-ESI-Q-Orbitrap analysis. Network pharmacology provides a new approach that allows researchers to systematically elucidate the inherent link between the active chemical compounds in TCMs and the therapeutic protein targets of a specific disease. Molecular docking is a crucial tool that can be used to evaluate the interaction between ligand and receptor through the principle of complementation of geometry, energy and chemical environment. It can also be used to propose structural hypotheses of the ligand–target interaction so that virtual screening of the biological activity of the compounds can be performed and their pharmacological mechanisms can be elucidated.

This study also carried out an in vivo animal experiment. High-fat diet (HFD) is an important factor in inducing insulin resistance, which is one of the main characteristics of T2DM. As a DNA alkylation reagent, streptozotocin (STZ) is more powerful and selective in damaging islet β cells and less harmful to the liver and kidney. An STZ-induced T2DM animal model can also better simulate the pathogenesis of human beings [6]. HFD and low-dose STZ stimulation in rats were used to mimic the metabolic characteristics of T2DM in this study. The pharmacodynamic indexes, biochemical indexes, histopathological tissues and hypoglycemic mechanisms of *D. nobile* in the T2DM rats were also analyzed. The above methods helped us to better explore the material basis and hypoglycemic pharmacological mechanisms of *D. nobile*, and to promote research and development of new hypoglycemic drugs.

## 2. Results and Discussion

### 2.1. Chemical Constituents of D. nobile

**Figure 1:**
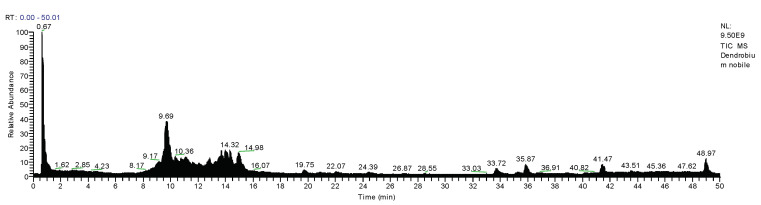
TIC chromatogram of *D. nobile* extract by UPLC-ESI-Q-Orbitrap.

Total ions currents (TICs) of all positive ions, negative ions, daughter ions of hypoglycemic DNE were collected by the data-dependent acquisition (DDA) detection module of UPLC-ESI-Q-Orbitrap (Figure 1). The molecular formulae from the detected quasi-molecular ion peaks with 5-decimal place mass-to-charge ratios were easily deduced by an accurate mass calculator, and a mass deviation (Diff) of less than 5 ppm and the nitrogen rule were also adopted herein; these preliminarily identified compounds were further confirmed by matching with the fragment ion information, chromatographic retention time and natural product databases. There were some compounds with the same molecular formula, which can be distinguished by their chemical polarity differences and literature reports. According to the methods described above, there are 39 natural compounds in *D. nobile* that had been finally identified, including alkaloids, bibenzyls, phenanthrenes and glycosides (Table 1).

#### 2.1.1. Alkaloids

**Figure 2:**
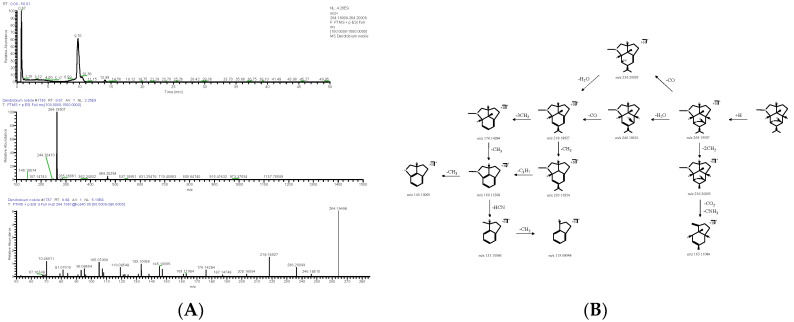
Extracted ion chromatogram: MS^1^ and MS^2^ spectra in positive ion mode (**A**), and proposed main fragments of dendrobine (DN 10) (**B**).

**Figure 3:**
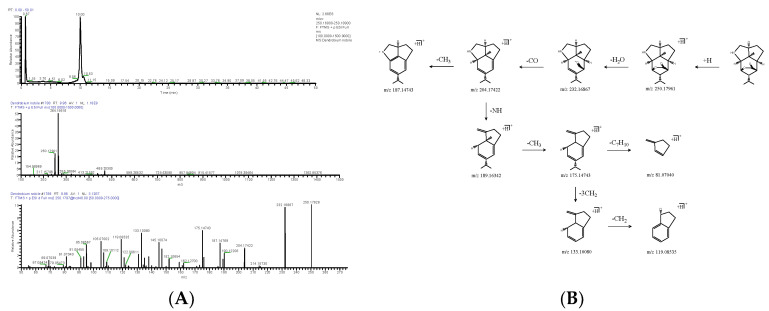
Extracted ion chromatogram: MS^1^ and MS^2^ spectra in positive ion mode (**A**), and proposed main fragments of nordendrobin (DN 11) (**B**).

Nine alkaloids were identified from DNE. Alkaloids in *D. nobile* responded well in the positive ion mode. The molecular formula of DN (compound from *D. nobile*) 10 was accurately predicted as C_16_H_25_NO_2_ by the quasi-molecular ion peak ([M+H]^+^) at *m*/*z* 264.19507 (ppm −2.784). The fragment ion *m*/*z* 236.20093 [M+H-2CH_2_]^+^ was produced by the loss of -2CH_2_ from [M+H]^+^, and the latter was further cleaved -CO_2_ and -CNH_3_ to obtain *m*/*z* 163.11084 [M+H-C_4_H_5_O_2_N]^+^; *m*/*z* 218.18927 [M+H-CH_2_O_2_]^+^ as a fragment ion was produced by the loss of -H_2_O and -CO from [M+H]^+^, and the latter was further cleaved -3CH_2_ and -CH_3_ to obtain *m*/*z* 176.14284 [M+H-C_4_H_4_O_2_]^+^ and *m*/*z* 203.16554 [M+H-C_2_H_5_O_2_]^+^; *m*/*z* 160.11208 [M+H-C_5_H_11_O_2_]^+^ was derived from *m*/*z* 176.14284 by the cleavage of -CH_3_, and the latter was further cleaved -CH_3_ and -HCN to obtain *m*/*z* 145.10065 [M+H-C_6_H_14_O_2_]^+^ and *m*/*z* 133.10066 [M+H-C_6_H_12_O_2_N]^+^; and the fragment ion *m*/*z* 133.10066 could drop -CH_2_ to produce *m*/*z* 119.08548 [M+H-C_7_H_14_O_2_N]^+^ (Figure 2). After the relevant research literature comparison [7,8], DN 10 was inferred as dendrobine (C_16_H_25_NO_2_). The molecular formula of DN 11 was C_15_H_23_NO_2_, as established by the [M+H]^+^ peak at *m*/*z* 250.17961 (ppm −2.181). The positive quasi-molecular ion *m*/*z* 250.17961 [M+H]^+^ was fragmented into fragment ion *m*/*z* 232.16867 [M+H-H_2_O]^+^ and was cleaved -CO to produce fragment ion *m*/*z* 204.17422 [M+H-CH_2_O_2_]^+^, the latter further lost -CH_3_ and -NH and obtained fragment ion *m*/*z* 187.14743 [M+H-C_2_H_5_O_2_]^+^ and *m*/*z* 189.16342 [M+H-CH_3_O_2_N]^+^; the fragment ion *m*/*z* 189.16342 lost -CH_3_ and obtained *m*/*z* 175.14743 [M+H-C_2_H_6_O_2_N]^+^, and the latter could drop -C_7_H_10_ and -3CH_2_ to produce *m*/*z* 81.07040 [M+H-C_9_H_16_O_2_N]^+^ and *m*/*z* 133.10080 [M+H-C_5_H_12_O_2_N]^+^; and *m*/*z* 119.08535 [M+H-C_6_H_14_O_2_N]^+^ as a fragment ion was produced by the loss of -CH_2_ from *m*/*z* 133.10080 (Figure 3). DN 11 was finally confirmed as nordendrobin (C_15_H_23_NO_2_).

#### 2.1.2. Bibenzyl

**Figure 4:**
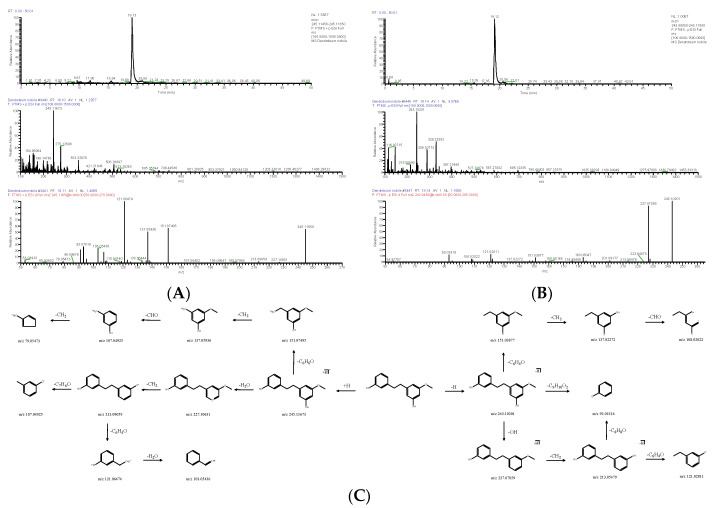
Extracted ion chromatogram: MS^1^ and MS^2^ spectra in positive ion mode (**A**) and negative ion mode (**B**), and proposed main fragments of batatasin III (DN 26) (**C**).

**Figure 5:**
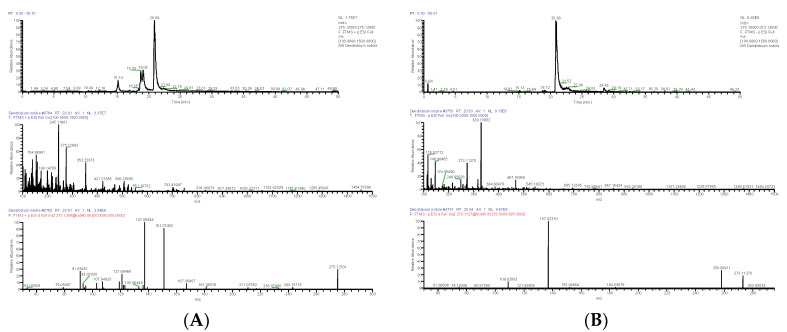

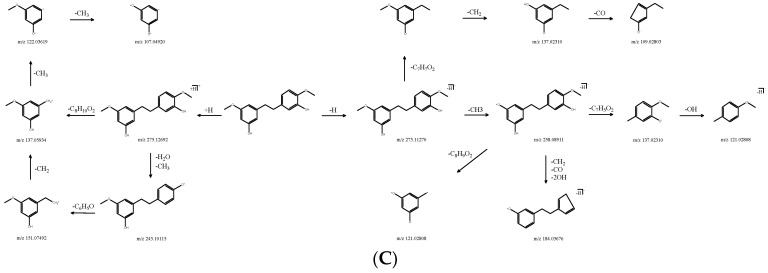
Extracted ion chromatogram: MS^1^ and MS^2^ spectra in positive ion mode (**A**) and negative ion mode (**B**), and proposed main fragments of gigantol (DN 29) (**C**).

There were five bibenzyls identified from DNE. The molecular formula of DN 26 was calculated to be C_15_H_16_O_3_, and its positive quasi-molecular ion *m*/*z* 245.11673 (ppm −2.003) [M+H]^+^ was fragmented into fragment ions *m*/*z* 151.07495 [M+H-C_6_H_6_O]^+^ and *m*/*z* 227.10631 [M+H-H_2_O]^+^. The fragment ion *m*/*z* 151.07495 successively discarded -CH_2_, -CHO, and -CH_2_, and fragment ions *m*/*z* 137.05936 [M+H-C_7_H_8_O]^+^, *m*/*z* 107.04925 [M+H-C_8_H_9_O_2_]^+^ and *m*/*z* 79.05473 [M+H-C_9_H_11_O_2_]^+^ were generated in turn. The fragment ion *m*/*z* 227.10631 could drop -CH_2_ to produce *m*/*z* 213.09059 [M+H-CH_4_O]^+^, and the latter further lost -C_7_H_6_O and -C_6_H_4_O to obtain *m*/*z* 107.04925 [M+H-C_8_H_10_O_2_]^+^ and *m*/*z* 121.06474 [M+H-C_7_H_8_O_2_]^+^; *m*/*z* 103.05436 [M+H-C_7_H_10_O_3_]^+^ was derived from *m*/*z* 121.06474 by the cleavage of -H_2_O. The negative quasi-molecular ion *m*/*z* 243.10201 (ppm 1.806) [M-H]^−^ was fragmented into fragment ions *m*/*z* 93.03316 [M-H-C_9_H_10_O_2_]^−^, *m*/*z* 151.03877 [M-H-C_6_H_4_O]^−^ and *m*/*z* 227.07059 [M-H-OH]^−^; *m*/*z* 137.02272 [M-H-C_7_H_6_O]^−^ as a fragment ion was produced by the loss of -CH_2_ from *m*/*z* 151.03877 (Figure 4), and the latter was further cleaved -CHO to obtain *m*/*z* 108.02022 [M-H-C_8_H_7_O_2_]^−^; *m*/*z* 213.05479 [M-H-CH_3_O]^−^ was derived from *m*/*z* 227.07059 by the cleavage of -CH_2_, and the latter was further cleaved -C_6_H_4_O to obtain *m*/*z* 121.02811 [M-H-C_7_H_7_O_2_]^−^. DN 26 was finally confirmed as batatasin III (C_15_H_16_O_3_). The molecular formula of DN 29 was C_16_H_18_O_4_, and its positive quasi-molecular ion *m*/*z* 275.12692 (ppm −3.146) [M+H]^+^ was fragmented into fragment ions *m*/*z* 137.05934 [M+H-C_8_H_10_O_2_]^+^ and *m*/*z* 243.10115 [M+H-CH_5_O]^+^. The fragment ion *m*/*z* 243.10115 successively discarded -C_6_H_5_O, -CH_2_, -CH_3_, and -CH_3_, and fragment ions *m*/*z* 151.07492 [M+H-C_7_H_10_O_2_]^+^, *m*/*z* 137.05943 [M+H-C_8_H_12_O_2_]^+^, *m*/*z* 122.03619 [M+H-C_9_H_15_O_2_]^+^ and *m*/*z* 107.04920 [M+H-C_10_H_18_O_2_]^+^ were generated in turn. The negative quasi-molecular ion *m*/*z* 273.11270 (ppm −2.819) [M-H]^−^ was fragmented into fragment ions *m*/*z* 137.02310 [M-H-C_8_H_9_O_2_]^−^ and *m*/*z* 258.08911 [M-H-CH_3_]^−^; *m*/*z* 109.02803 [M-H-C_9_H_9_O_3_]^−^ as a fragment ion was produced by the loss of -CO from *m*/*z* 137.02310 (Figure 5); the fragment ion *m*/*z* 258.08911 could respectively drop -C_8_H_9_O_2_, (-CH3, -CO and -2OH), and -C_7_H_5_O_2_ to produce *m*/*z* 121.02808 [M-H-C_9_H_12_O_2_]^−^, *m*/*z* 184.03676 [M-H-C_3_H_7_O_3_]^−^ and *m*/*z* 137.02310 [M-H-C_8_H_8_O_2_]^−^, and the latter further lost -OH to obtain *m*/*z* 121.02808. DN 29 was finally inferred as gigantol (C_16_H_18_O_4_).

#### 2.1.3. Phenanthrene

**Figure 6:**
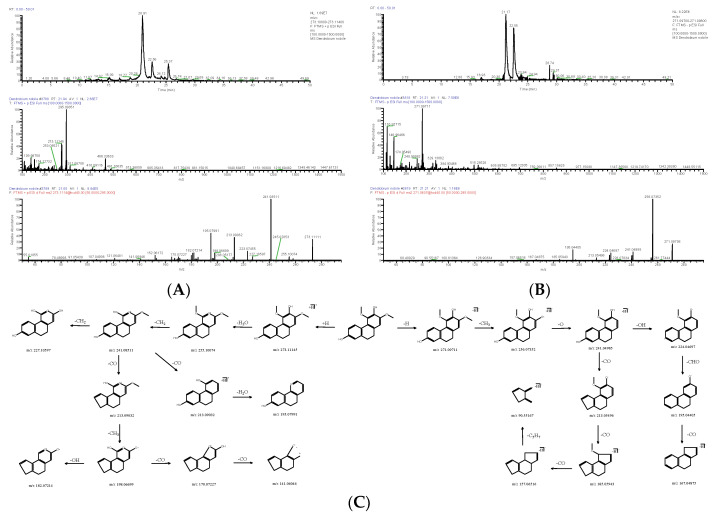
Extracted ion chromatogram: MS^1^ and MS^2^ spectra in positive ion mode (**A**) and negative ion mode (**B**), and proposed main fragments of ephemeranthol B (DN 30) (**C**).

**Figure 7:**
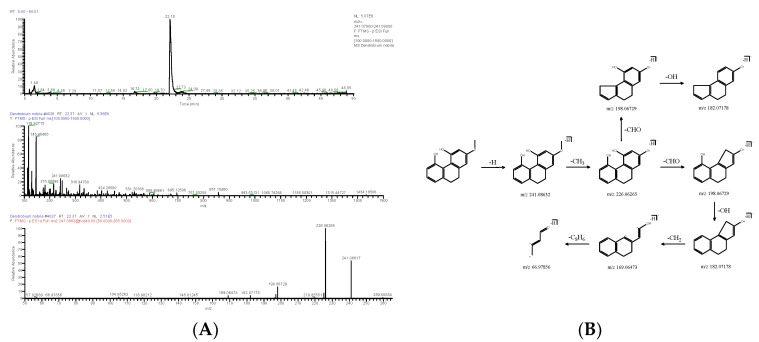
Extracted ion chromatogram: MS^1^ and MS^2^ spectra in negative ion mode (**A**), and proposed main fragments of 2-methoxy-9,10-dihydrophenanthrene-4,5-diol (DN 32) (**B**).

Phenanthrene was identified from DNE. The molecular formula of DN 30 was C_16_H_16_O_4_, as indicated by the positive quasi-molecular ion peak ([M+H]^+^) at *m*/*z* 273.11145 (ppm −2.510); *m*/*z* 255.10074 [M+H-H_2_O]^+^ as a fragment ion was produced by the loss of -H_2_O from [M+H]^+^, and the latter was further cleaved -CH_2_ to obtain *m*/*z* 241.08511 [M+H-CH_4_O]^+^, which then could generate *m*/*z* 227.10597 [M+H-C_2_H_6_O]^+^, *m*/*z* 213.09032 [M+H-C_2_H_4_O_2_]^+^ and *m*/*z* 195.07991 [M+H-C_2_H_6_O_3_]^+^. The fragment ion *m*/*z* 198.06699 [M+H-C_3_H_7_O_2_]^+^ was produced by the loss of -CH_3_ from *m*/*z* 213.09032, and it could drop -OH and -CO to produce *m*/*z* 182.07214 [M+H-C_3_H_8_O_3_]^+^ and *m*/*z* 170.07227 [M+H-C_4_H_7_O_3_]^+^; the latter further lost -CO to obtain *m*/*z* 141.06046 [M+H-C_5_H_7_O_4_]^+^. The negative quasi-molecular ion *m*/*z* 271.09711 (ppm 2.304) [M-H]^−^ was successively fragmented into fragment ions *m*/*z* 256.07352 [M-H-CH_3_]^−^, *m*/*z* 241.04985 [M-H-CH_4_O]^−^, *m*/*z* 224.04697 [M-H-CH_5_O_2_]^−^, *m*/*z* 195.04405 [M-H-C_2_H_6_O_3_]^−^ and *m*/*z* 167.04875 [M-H-C_3_H_6_O_4_]^−^ in turn by the loss of -CH_3_, -OH, -OH, -CHO and -CO (Figure 6). The fragment ion *m*/*z* 241.04985 could successively drop -CO, -CO, CO and -C_5_H_7_ to obtain *m*/*z* 213.05496 [M-H-C_2_H_4_O_2_]^−^, *m*/*z* 185.05943 [M-H-C_3_H_4_O_3_]^−^, *m*/*z* 157.06516 [M-H-C_4_H_4_O_4_]^−^ and *m*/*z* 90.55167 [M-H-C_9_H_11_O_4_]^−^ in turn. DN 30 was finally confirmed as ephemeranthol B (C_16_H_16_O_4_). The molecular formula of DN 32 was predicted as C_15_H_14_O_3_ based on the [M-H]^−^ peak at *m*/*z* 241.08632 (ppm 1.656). The negative quasi-molecular ion *m*/*z* 241.08617 [M-H]^−^ could successively generate fragment ions *m*/*z* 226.06265 [M-H-CH_3_]^−^, *m*/*z* 198.06729 [M-H-C_2_H_4_O]^−^, *m*/*z* 182.07178 [M-H-C_2_H_5_O_2_]^−^, *m*/*z* 169.06473 [M-H-C_3_H_7_O_2_]^−^ and *m*/*z* 66.97856 [M-H-C_11_H_13_O_2_]^−^ by the loss of -CH_3_, -CHO, -OH, -CH_2_ and -C_8_H_6_ (Figure 7). DN 32 was finally confirmed as 2-methoxy-9,10-dihydrophenanthrene-4,5-diol (C_15_H_14_O_3_).

#### 2.1.4. Other

**Figure 8:**
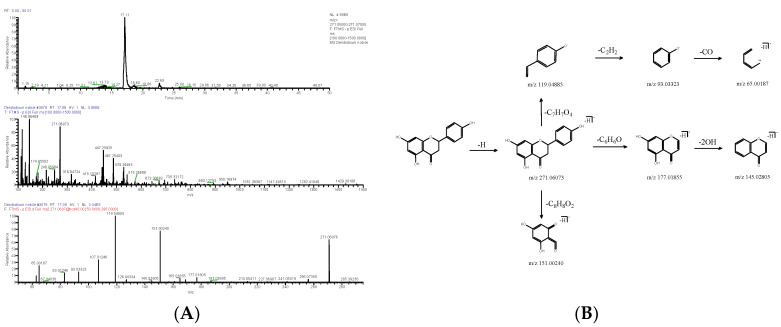
Extracted ion chromatogram: MS^1^ and MS^2^ spectra in negative ion mode (**A**), and proposed main fragments of naringenin (DN 23) (**B**).

Other compounds included flavonoids, glycosides and so on. The molecular formula of DN 23 was C_15_H_12_O_5_, and its negative quasi-molecular ion *m*/*z* 271.06073 (ppm 2.324) [M-H]^−^ was fragmented into fragment ions *m*/*z* 119.04885 [M-H-C_7_H_7_O_4_]^−^, *m*/*z* 177.01855 [M-H-C_6_H_6_O]^−^ and *m*/*z* 151.00240 [M-H-C_8_H_8_O_2_]^−^.The fragment ion *m*/*z* 119.04885 could drop -C_2_H_2_ to produce *m*/*z* 93.03323. The latter further lost -CO and obtained *m*/*z* 65.00187. *m*/*z* 145.02805 [M-H-C_6_H_8_O_3_]^−^ as a fragment ion was produced by the loss of -2OH from *m*/*z* 177.01855 (Figure 8). After the relevant research literature comparison [9], DN 23 was identified as naringenin (C_15_H_12_O_5_).

### 2.2. Hypoglycemic Effects of D. nobile

#### 2.2.1. Effects of *D. nobile* on Conventional Pharmacodynamic Indexes

**Figure 9:**
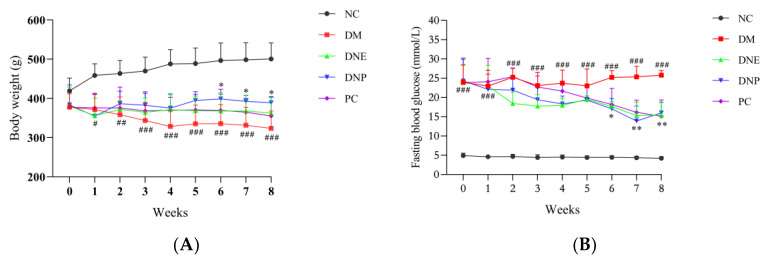
Effects of *D. nobile* on body weight (**A**) and FBG (**B**) of T2DM rats. All data are expressed as mean ± S.D. (n = 6 per group). Compared with the NC group, ^#^ *p* < 0.05, ^##^ *p* < 0.01, and ^###^ *p* < 0.001; compared with the DM group, * *p* < 0.05 and ** *p* < 0.01.

The T2DM rats in the DM group showed significant body weight loss and abnormally elevated blood glucose than those of the NC group; the rats in each drug treatment group showed different degrees of improvement (*p* < 0.05) (Figure 9), with the DNP group showing more effective effects than DNE group and the PC group. The results indicated that *D. nobile* had a beneficial effect on mitigating body weight loss and regulating serum glucose in T2DM rats.

**Figure 10:**
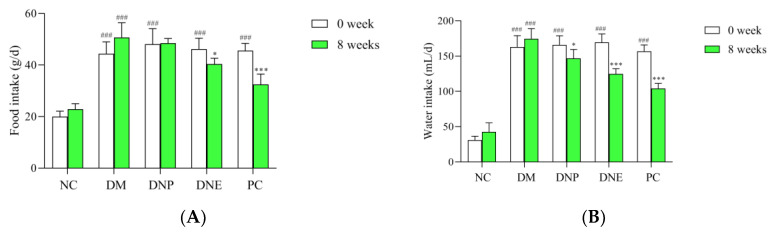
Effects of *D. nobile* on food intake (**A**) and water intake (**B**) of T2DM rats. All data are expressed as mean ± S.D. (n = 6 per group). Compared with the NC group, ^###^ *p* < 0.001; compared with the DM group, * *p* < 0.05 and *** p < 0.001.

Polydipsia and polyphagia are the significant features of T2DM, and these were evident in the DM group, with the food intake and water intake in the DM group being significantly increased. DNE, DNP and metformin as the positive control drugs could significantly decrease water intake and food intake of the T2DM rats (*p* < 0.05) (Figure 10). The results showed that *D. nobile* could improve polydipsia and polyphagia in the T2DM rats.

**Figure 11:**
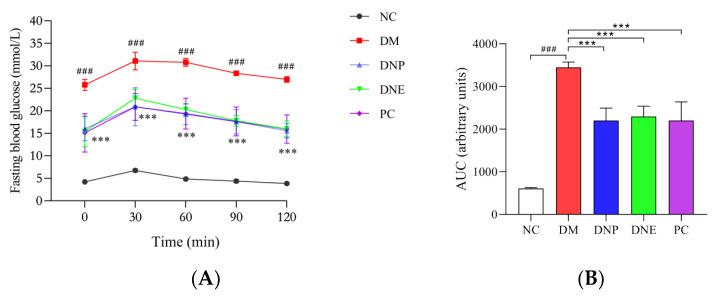
Effect of *D. nobile* on oral glucose tolerance of T2DM rats: blood glucose levels (**A**) and their AUCs (**B**). All data are expressed as mean ± S.D. (n = 6 per group). Compared with the NC group, ^###^ *p* < 0.001; compared with the DM group, *** *p* < 0.001.

Basal blood glucose levels of the T2DM rats were already at the highest; blood glucose levels rose rapidly after orally administered glucose to the T2DM rats in the OGTT, remained at high values, and did not return to the original levels within 120 min. The rats treated with DNE, DNP or metformin had relatively low blood glucose levels (Figure 11), The AUCs of blood glucose levels can better show the hypoglycemic effect of DNP and PC (*p* < 0.001). The OGTT results indicated *D. nobile* could ameliorate glucose intolerance in T2DM rats.

**Figure 12:**
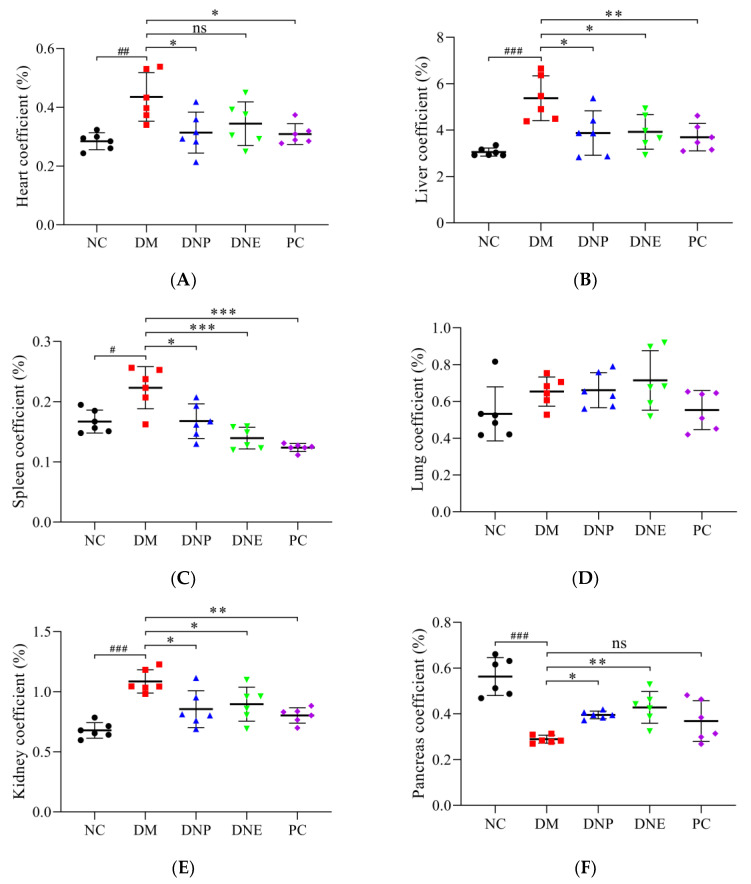
Effects of *D. nobile* on organ coefficients ((**A**): heart, (**B**): liver, (**C**): spleen, (**D**): lung, (**E**): kidney and (**F**): pancreas) of T2DM rats. All data are expressed as mean ± S.D. (n = 6 per group). Compared with the NC group, ^#^ *p* < 0.05, ^##^ *p* < 0.01, and ^###^ *p* < 0.001; compared with the DM group, * *p* < 0.05, ** *p* < 0.01, and *** *p* < 0.001.

The organ coefficients of the heart, liver, spleen and kidney in the DM group were increased, except that pancreas coefficient was decreased (Table 2), showing DNE and DNP can effectively improve the above organ coefficients (*p* < 0.05). Lung coefficient did not show statistical differences between the different groups. The final body weight of the DM group at the end of the experiment was lower than the NC group and the drug-treated groups (Figure 12).

#### 2.2.2. Effects of *D. nobile* on Biochemical Indexes

**Figure 13:**
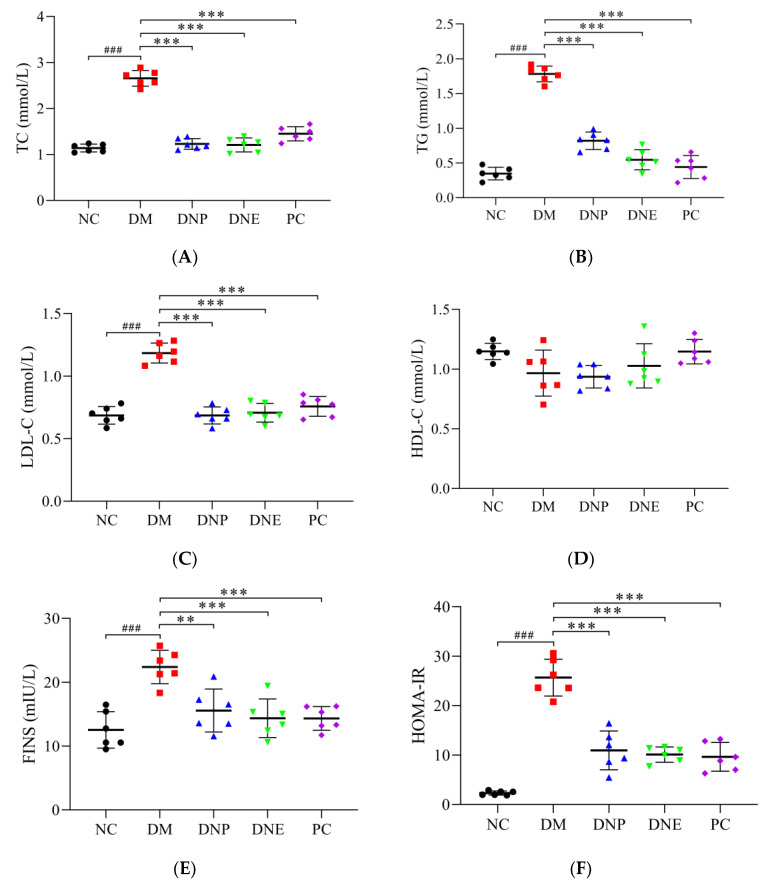

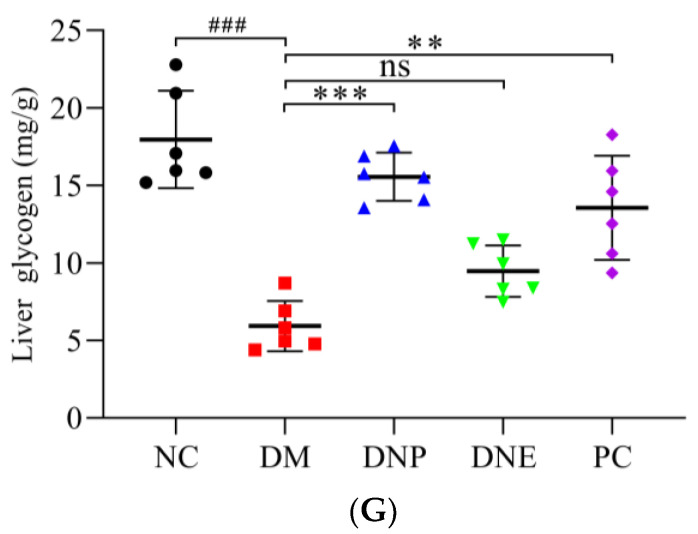
Effects of *D. nobile* on serum lipid profile, insulin level and liver glycogen ((**A**): TC, (**B**): TG, (**C**): LDL-C, (**D**): HDL-C, (**E**): FINS, (**F**): HOMA-IR and (**G**): liver glycogen) contents of T2DM rats. All data are expressed as mean ± S.D. (n = 6 per group). Compared with the NC group, ^###^ *p* < 0.001; compared with the DM group, ** *p* < 0.01 and *** *p* < 0.001.

*D. nobile* could regulate blood lipid profile, reduce insulin levels and increase liver glycogen synthesis in T2DM rats. The serum levels of TC, TG, LDL-C, FINS and HOMA-IR in the DM group increased significantly compared to those of the NC group (Table 3), while liver glycogen content was significantly decreased (*p* < 0.001). The TC, TG, LDL-C, FINS and HOMA-IR levels of the DNE and DNP groups were all significantly reduced (*p* < 0.001) compared to those of the DM group; liver glycogen contents under the treatment of DNE and DNP were significantly increased (*p* < 0.001); and there were no changes in the HDL-C levels between these groups (Figure 13). Therefore, these results suggested that *D. nobile* ameliorated hyperlipidemia and insulin resistance in T2DM rats, and it reduced blood glucose concentration by increasing liver glycogen storage.

#### 2.2.3. Effects of *D. nobile* on Histopathological Tissues

**Figure 14:**
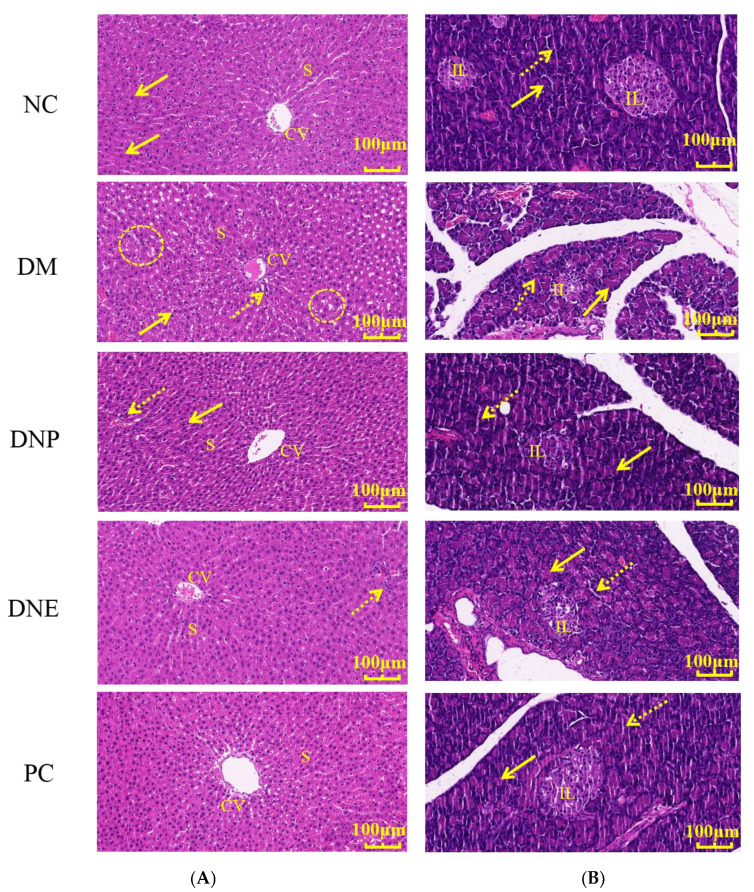
Effects of *D. nobile* on liver (**A**) and pancreas (**B**) tissues of T2DM rats. Central vein (CV), hepatic sinusoid (s), hepatocytes (arrow), inflammatory cell infiltration (dotted arrow) and accumulation of fatty infiltration (circle) in the liver, islets of Langerhans (IL), acinar structure (arrow) and duct (dotted arrow) in the pancreas.

The H&E-stained features of normal rat hepatic tissue generally contained regular-shaped hepatic lobules, radial distribution of hepatic cords and hepatic sinusoids around the central vein, polygonal and intact hepatocyte morphology, orderly arranged hepatocytes, and circular hepatocyte nuclei. The T2DM rats in the DM group had disordered hepatic cords, irregular arrangement of hepatocytes with steatosis, distorted and narrow hepatic sinusoids, and infiltration of inflammatory cells around the central vein (Figure 14), showing the liver tissue was seriously disordered with fat infiltration and accumulation. In the DNE and DNP groups, the morphology of hepatic lobules and hepatocytes was basically restored to normal, and hepatic cords and hepatic sinusoids were significant, but there was only a little fat deposition and inflammatory cell infiltration. The PC group also showed a similar effect, and there was no apparent inflammatory cell infiltration.

The rats’ pancreatic tissues stained with H&E were analyzed; normal rat pancreatic tissues were intact, and the islets of Langerhans were abundant, oval and complete, being uniformly distributed by islet cells. The islets of Langerhans having shallower cytoplasm than the surrounding acinar cells were clearly demarcated from the exocrine glands; the ducts of exocrine were arranged neatly; and the acinar cells were clear. The endocrine and exocrine glands of the T2DM rats underwent degeneration, and the number and area of islets of Langerhans were decreased, showing irregular clusters with vacuole infiltration. There were few islet cells, which were non-uniformly distributed and free at the edge of the islet of Langerhans; the boundary of the exocrine glands was incomplete; and damaged acinar cells and disorderly arranged ducts were also common. The above-described histopathological features were improved after medical treatment with DNE and DNP, the conduits were arranged neatly without obvious vacuole infiltration (Figure 14), and some clusters of islet cells and mild degeneration of exocrine glands still existed.

### 2.3. Hypoglycemic Mechanism of D. nobile

#### 2.3.1. Network Pharmacological Analysis

**Figure 15:**
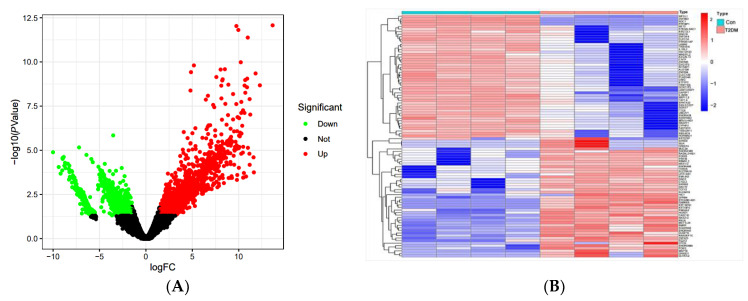
The volcano map (**A**) and heat map (**B**) of DEGs in T2DM patients. Red represents up-regulated genes, green represents down-regulated genes, and black represents unchanged genes.

A total of 32 compounds were adopted from the 39 compounds identified in DNE according to the ADME principle of DL and GIA, and 806 potential targets were predicted based on the chemical structures of these 32 compounds in *D. nobile*. A total of 20 samples from 10 control human plasma and 10 T2DM samples were obtained from the GSE153315 dataset; the DEGs between the T2DM and normal samples were defined by the limma packet, and a volcanic map of the DEGs shows that a total of 2815 DEGs were obtained, including 1282 up-regulated genes and 1533 down-regulated genes (Figure 15). The clustering heat map of DEGs is able to distinguish the T2DM group and the normal group. There was a total of 5198 T2DM-related target genes collected from the GeneCards databases, and 75 common target genes were finally obtained through the Venn diagram. Furthermore, the common target genes were uploaded to the STRING database for analysis with a confidence score ≥ 0.4, the PPI network had 70 nodes, and 288 edges in total were visualized by Cytoscape 3.7.0. The node size and color depth represent high degree values, and the importance of the targets is positively correlated with the degree value.

**Figure 16:**
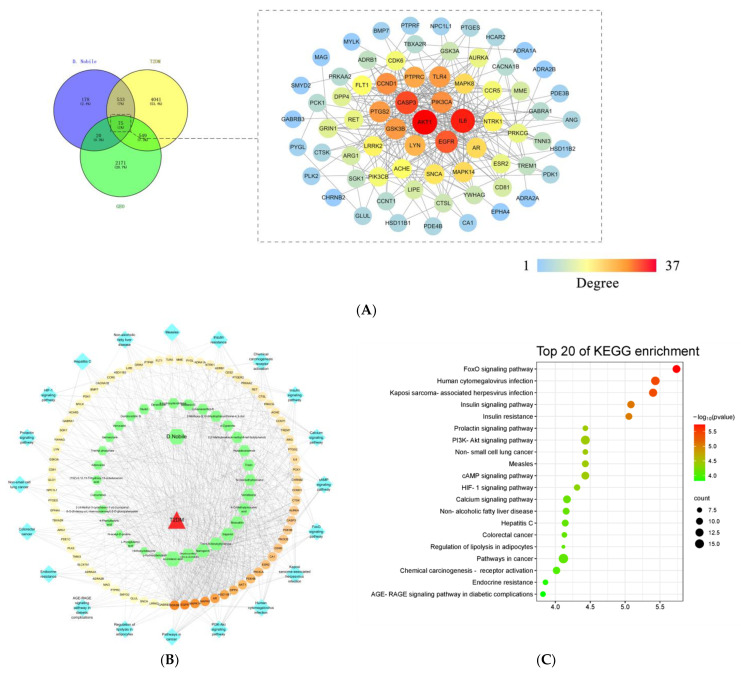
PPI network (**A**), “Herb-Compounds-Targets-Pathway-Disease” network (**B**), and bubble diagram of the KEGG enrichment analysis (**C**) of *D. nobile* in the treatment of T2DM after the network pharmacological analysis.

After topological analysis, the top 10 targets were screened from the PPI network, including AKT1, IL6, CASP3, EGFR, CCND1, PIK3CA, GSK3B, PTGS2, TLR4 and PTPRC. The “Herb-Compounds-Targets-Pathway-Disease” network of *D. nobile* contains 129 nodes and 814 edges (Figure 16). The top 10 compounds were identified as key active compounds according to the degree value, including α-linolenic acid, dihydroconiferyl dihydro-p-coumarate, naringenin, *trans-N*-feruloyltyramine, gigantol, moscatilin, 4-*O*-methylpinosylvic acid, venlafaxine, nordendrobin and tristin. The top 10 targets were also screened out, including GSK3B, EGFR, MAPK14, MAPK8, AR, HSD11B1, DPP4, AKT1, PDE4B and PIK3CA. AKT1, PIK3CA, GSK3B and EGFR are the common targets of the PPI network and “Herb-Component-Targets-Pathway-Disease” network, which may be the core targets of hypoglycemic *D. nobile*. The KEGG enrichment analysis showed that *D. nobile* can treat T2DM through the synergistic action of multiple pathways, mainly involving in the pathogenesis of diabetic metabolic syndrome and insulin resistance through the PI3K-AKT signaling pathway, the FOXO signaling pathway, the AGE-RAGE signaling pathway in diabetic complications, and the insulin signaling pathway (Figure 16).

#### 2.3.2. Molecular Docking Analysis

**Figure 17:**
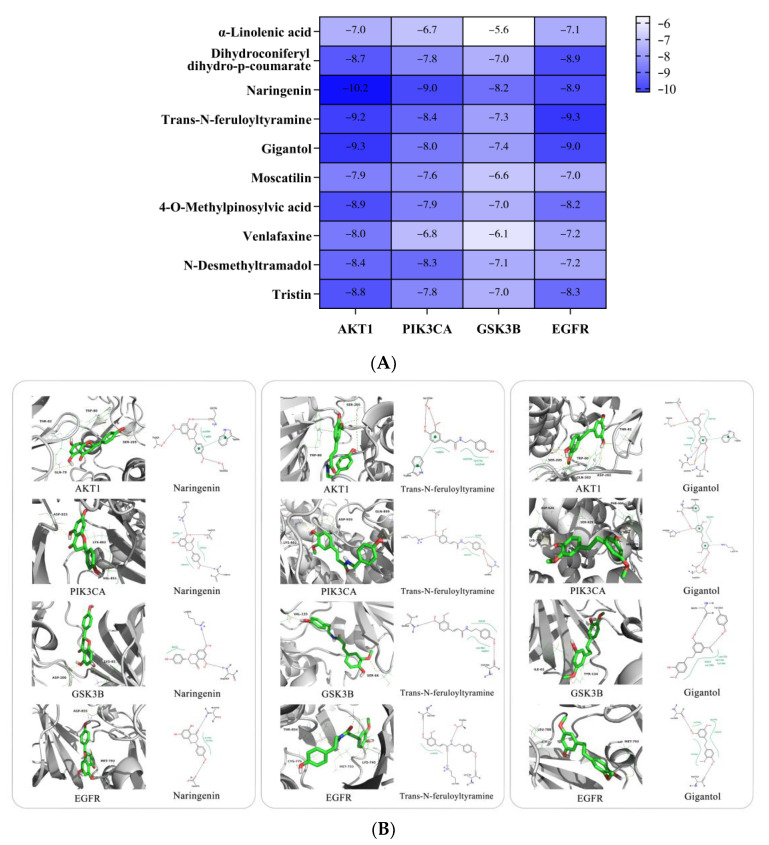
Molecular docking binding energy between key active compounds of *D. nobile* and core targets (**A**), and molecular docking model of naringenin, *trans*-N-feruloyltyramine, gigantol with AKT1, PIK3CA, GSK3B and EGFR (**B**).

Molecular docking was performed in this study to further explore the binding affinity between the key active compounds and the core targets; a binding affinity below −5.0 kcal/mol indicates the binding activity of the ligand molecules and receptor proteins is good, while affinities below −7.0 kcal/mol indicate a stronger binding activity [10,11]. The values of the binding energy between the key active compounds of *D. nobile* and the core targets indicate well-binding affinity, and the 2D and 3D docking structures in the lowest binding energy are displayed (Figure 17); a positive correlation is between the depth of the blue color and compound–target binding activity, and the major binding interactions mainly contain hydrogen bonding and hydrophobic interactions. The naringenin–AKT1 docking was chosen as an example to elucidate their interactions, and the binding energy was identified as −10.2 kcal/mol; naringenin could form intermolecular hydrogen bonds with amino acid residues, including Trp79A, Trp82A and Ser205A of AKT1 protein. Moreover, π-π accumulation and hydrophobic interaction between naringenin and amino acid residue Trp80A of AKT1 protein were also observed.

#### 2.3.3. Insulin Signaling Pathway

The key proteins related to insulin signal transduction, glycogen synthesis and gluconeogenesis in the liver were analyzed to perform an in vivo experimental verification; the PI3K/Akt pathway as a key component of insulin signaling pathway was considered to be essential for glucose transport. The protein levels of p-PI3K, p-PI3K/PI3K, p-AKT and p-AKT/AKT in the liver tissue of the DM rats were significantly decreased (*p* < 0.05) compared to the NC rats, and the protein levels of p-PI3K, p-PI3K/PI3K, p-AKT and p-AKT/AKT in the liver tissue of the rats under the treatment of DNE and DNP were significantly increased (*p* < 0.05). These results indicated *D. nobile* may become a potential therapeutic agent by activating the PI3K/Akt signaling pathway. 

**Figure 18:**
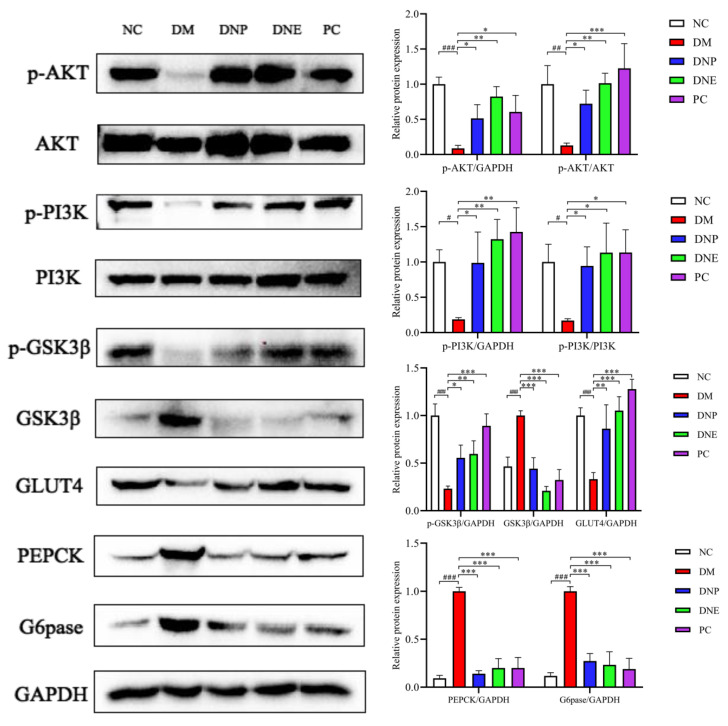
Effects of *D. nobile* on the expression of P-PI3K, P-PI3K/PI3K, P-Akt, P-Akt/Akt, PEPCK, G6Pase, p-GSK3β, GSK3β and GLUT4 proteins in the liver of T2DM rats. Compared with the NC group, ^#^ *p* < 0.001, ^##^ *p* < 0.001, and ^###^ *p* < 0.001; compared with the DM group, * *p* < 0.01, ** *p* < 0.01, and *** *p* < 0.001.

Gluconeogenesis and glycogenolysis are two major pathways for endogenous glucose production, and PEPCK and G6Pase are the rate-limiting enzymes to control gluconeogenesis in the liver, while GSK3β is the key enzyme involved in glycogen synthesis. In addition, the liver can increase tissue uptake of glucose by promoting the translocation of GLUT4 to the plasma membrane. The protein levels of PEPCK, G6Pase and GSK3β in the liver tissue were significantly increased in the DM rats (*p* < 0.001) compared to the NC rats, while the protein levels of PEPCK, g6pase and GSK3β in the DNP, DNE and PC rats were decreased significantly (*p* < 0.001). Furthermore, the protein levels of p-GSK3β and GLUT4 in the liver tissue were significantly decreased in the DM rats compared to the NC rats (*p* < 0.001), and the protein levels of p-GSK3β and GLUT4 were remarkably increased in the DNP and DNE rats (*p* < 0.05) (Figure 18). These results indicated that *D. nobile* promoted liver glycogen synthesis and inhibited liver gluconeogenesis to reduce serum glucose levels.

The UPLC-ESI-Q-Orbitrap MS analysis was carried out to elucidate chemical constituents of *D. nobile*, and 39 natural compounds were identified, including alkaloids, bibenzyls, phenanthrenes, glycosides and others. Total alkaloids of *D. nobile* had been reported to have a blood glucose-lowering effect on T2DM rats, reduce hepatic steatosis [3] and protect islet cells [12]. Phenols, dibenzyls, phenanthrenes and flavonoids of *D. nobile* have the potential to inhibit free radical accumulation, which usually leads to chronic hyperglycemia. α-linolenic acid, dihydroconiferyl dihydro-p-coumarate, naringenin, *trans-N*-feruloyltyramine, gigantol, moscatilin, 4-*O*-methylpinosylvic acid, venlafaxine, nordendrobin and tristin are regarded as the key active compounds of *D. nobile*. *Trans-N*-feruloyltyramine, venlafaxine and nordendrobin are alkaloid compounds, while naringenin, gigantol, moscatilin, 4-*O*-methylpinosylvic acid and tristin belong to phenolic compounds. α-Linolenic acid can prevent and treat T2DM by reducing inflammatory response and improving the abnormalities of lipid spectrum [13], and it can alleviate retinopathy of diabetic rats through anti-inflammatory and anti-oxidative stress [14]. Naringenin is a flavonoid compound with anti-inflammatory, anti-oxidative, and anti-tumor properties; it scavenges free radicals, reduces blood lipid, and can treat T2DM by improving glucose lipid metabolism [15] and inhibiting α-glucosidase activity [16]. *Trans-N*-feruloyltyramine has potential hypoglycemic effect because of its anti-oxidative stress effects [17] and inhibition of α-glucosidase activity [18]. Venlafaxine can improve painful peripheral diabetic neuropathy among T2DM patients [19], and gigantol can correct abnormal body weight and blood glucose of diabetic mice and treat retinopathy by inhibiting the expression of inflammatory factors TNF-α, IL-1β and IL-6 in the MAPK pathway [20]. Moscatilin was used for diabetes and asserted its effects by inhibiting the AGE-RAGE signaling pathway [21], which was closely related to inflammatory reactions and oxidative stress in diabetic wounds; moscatilin can promote the uptake of glucose by peripheral tissues or cells [22]. All these compounds in *D. nobile* had been proven to treat diabetes and relieve the symptoms of related diseases. The in vivo animal experiment proved that DNP had good hypoglycemic activity in this study. A study on the chemical structure of DNP will be performed in subsequent research work because of its complex stereostructure and large molecular weight.

A HFD/STZ-induced T2DM rat model was successfully established in this study with an increase in hyperglycemia and insulin resistance. *D. nobile* could significantly ameliorate body weight, insulin resistance, hyperglycemia and dyslipidemia in the T2DM rats. The food intake and water consumption of the T2DM rats increased, and their body weight decreased significantly, which was similar to the symptoms of polyphagia and weight loss of diabetic patients [23]. The oral administration of *D. nobile* could significantly improve the weight abnormality of the T2DM rats and normalize food intake and water consumption. T2DM is not only related to hyperglycemia but also to hyperinsulinemia and insulin resistance [24]. The fasting blood glucose and insulin levels in the HFD/STZ-induced T2DM rats were increased, and long-term hyperglycemia required more insulin, which led to a decrease in insulin sensitivity and insulin resistance. After eight weeks of intervention by *D. nobile*, FBG and insulin in the model rats significantly reduced, and the HOMA-IR index based on FBG and insulin was calculated; the results indicate that *D. nobile* can increase insulin sensitivity and reduce insulin resistance. Liver is an important insulin-targeting organ and the main site of glucose metabolism, which is responsible for regulating glucose and maintaining glucose homeostasis through gluconeogenesis and glycogen synthesis [25]; the amount of glycogen in the liver is an indirect reflection of insulin activity. The glycogen content of the treated group increased significantly, while the glycogen content of the T2DM rats decreased substantially in this study, which indicated that *D. nobile* could regulate glucose level by enhancing glycogen production. Insulin resistance is the main cause of the disorder of glucose and lipid metabolism in T2DM. The liver index of the T2DM rats increased significantly due to the accumulation of liver fat and the development of fatty liver [26], and this result was confirmed by the histopathological features of the liver, which showed that the levels of TC, TG and LDL-C in the DNE and DNP groups were significantly reduced, indicating that *D. nobile* can greatly improve hepatic steatosis and fatty liver, and substantially accelerate the clearance of cholesterol from peripheral tissues to the liver for catabolism.

**Figure 19:**
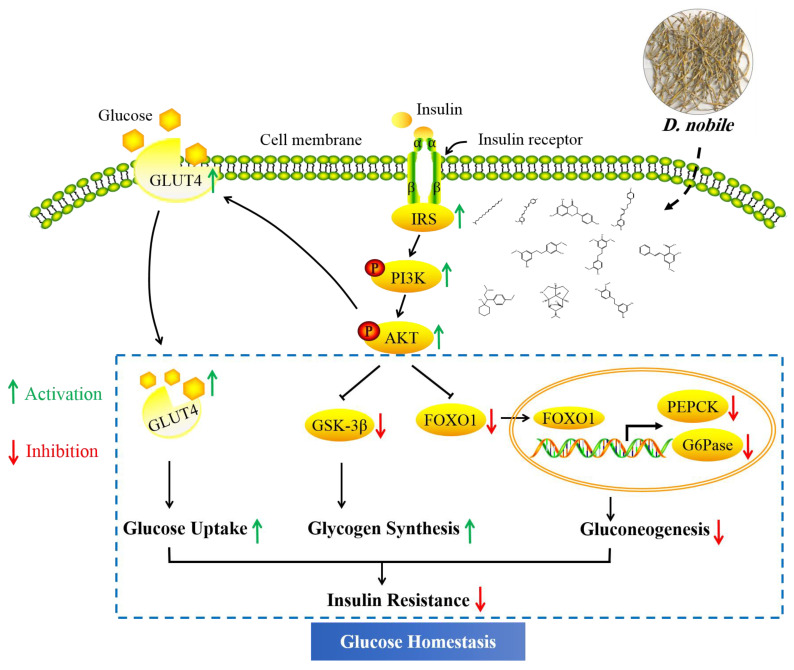
The hypoglycemic mechanism of *D. nobile* in the treatment of T2DM rats.

Almost all core targets of *D. nobile* were involved in the PI3K/AKT signaling pathway, the insulin signaling pathway, the FOXO signaling pathway and insulin resistance. AKT1, PIK3CA, GSK3B and EGFR might play an important role in the biological process of *D. nobile*, thereby intervening in type 2 diabetes. AKT1 is a serine/threonine protein kinase that improves β-cell function and glucose homeostasis [27]. PI3K family has also been confirmed to be involved in glucose regulation in the occurrence and development of diabetes. The activation of PI3K can induce insulin-related regulatory factors to regulate glucose metabolism. The PI3CA gene is responsible for coding the catalytic subunit of PI3K, and biological function of PI3CA involves regulating immunity, inflammatory process and the development of diabetes [28]. The inhibition of EGFR can delay the occurrence and development of diabetes and its complications, and EGFR has been reported to help the improvement of heart damage and remodeling by reducing oxidative stress in STZ-induced diabetic mice [29]. Insulin resistance is a crucial factor in the occurrence of T2DM, and its pathological state involves abnormalities in the insulin signaling pathway. When insulin binds to insulin receptors, insulin signal is transmitted from extracellular domain to intracellular domain to activate insulin receptor, and activated insulin receptor can be combined with insulin receptor substrate (IRS), thus activating the downstream PI3K/AKT pathway [30]. The PI3K/Akt signal pathway is one of the main signal transduction pathways that affects insulin and plays an important role in regulating glucose metabolism [31], including glycogen synthesis, promotion of glucose uptake and inhibition of hepatic gluconeogenesis in the liver. However, these liver processes are dysfunctional in T2DM as the liver becomes insulin resistant. The results showed that *D. nobile* might activate the insulin signaling pathway by up-regulating the protein expression levels of p-PI3K, p-PI3K/PI3K, p-AKT and p-AKT/AKT in diabetic rats, thereby improving the glucose metabolism disorder (Figure 19). It is well known that GSK3β, GLUT4 and FOXO1 are the key proteins regulated by phosphorylated AKT, and GSK3β plays an important role in regulating glycogen synthesis as a key negative regulator pathway of insulin signaling [32]. In this regard, *D. nobile* increased p-GSK3b levels and decreased GSK3B values, thereby regulating glucose metabolism by affecting glycogen synthesis. GLUT4 is a major insulin-reactive glucose transporter, which plays a key role in regulating whole-body glucose homeostasis. When carbohydrates are ingested, insulin activates the signaling cascade to stimulate GLUT4, which rapidly translocates to the plasma membrane, thereby promoting tissue uptake and utilization of glucose [33]. *D. nobile* promoted GLUT4 translocation and stimulated glucose uptake. On the other hand, PEPCK and G6Pase are rate-limiting enzymes that control liver gluconeogenesis and play an irreversible role in the gluconeogenesis pathway as the regulatory factors downstream of FOXO1 [34]. *D. nobile* can reduce liver gluconeogenesis by down-regulating the protein levels of PEPCK and G6Pase in T2DM rats, improve raw glucose level and glucose uptake, and relieve insulin resistance by restoring the insulin signaling pathway. However, further studies are needed to explore the relationship between the PI3K/Akt signaling pathway and glucose metabolism-related proteins.

## 3. Materials and Method

### 3.1. Chemicals and Reagents

Fresh *D. nobile* originated from Guizhou province and was supplied by Guizhou Wanshuntang Pharmaceutical Co., Ltd. in Guiyang in May 2021. This was identified by Associate Professor Shaohuan Liu from the Department of Pharmacognosy, School of Pharmacy, Guizhou Medical University.

PI3K (60225-1-Ig), AKT (10176-2-AP), Phospho-AKT (Ser473, 66444-1-Ig), GSK3β (22104-1-AP), Phospho-GSK3β (Ser9, 67558-1-Ig), GLUT4 (66846-1-Ig), PEPCK (16754-1-AP) and GAPDH (10494-1-AP) were purchased from Proteintech (Wuhan, China); G6pase (bs-21524R) was purchased from Bioss Biotechnology (Beijing, China); Phospho-PI3K (Y467/Y199, BS4605) was purchased from Bioworld Technology (Nanjing, China). Streptozocin (STZ, 830F022, Solarbio, Beijing, China), acetonitrile, methanol (HPLC-pure, Merck, Germany), formic acid (HPLC-pure, Sinopharm, Shanghai, China), ethanol, acetone (Analytically pure, Sinopharm, China) and pure water (Watsons, Guangzhou, China) were also used.

### 3.2. Sample Preparation

Fresh *D. nobile* was dried by natural light; the dried stem of *D. nobile* was pulverized into powder using a homogenous 24 mesh screen before the experiment, and then extracted 3 times by reflux extraction for 2 h each time, with 95% ethanol solution being as the extraction solvent. The reflux extract solution was collected and concentrated in the reduced-pressure vacuum state to a nonalcoholic solution. The *D. nobile* extract (DNE) was finally prepared for HRMS analysis and in vivo hypoglycemic experiment in the rats. After reflux extraction, the filtered residue of *D. nobile* was further extracted by water decoction and purified by the ethanol and acetone precipitation method to obtain *D. nobile* polysaccharide (DNP).

### 3.3. Qualitative Analysis of D. nobile

A total of 1.5 mg/mL of the DNE sample solution was dissolved in methanol and filtered by 0.22 μm lipophilic microporous filters before the HRMS analysis. UPLC-ESI-Q-Orbitrap (Thermo, Waltham, MA, USA), including a Dionex Ultimate 3000 UPLC and Thermo Scientific Q Exactive Orbitrap equipped with an electrospray ionization source (ESI), was applied for the qualitative analysis of *D. nobile*. An ACE Ultracore SuperC18 column (100 × 2.1 mm, 2.5 μm) with a temperature of 40 °C was used for chromatographic separation; the mobile phases consisted of 0.1% formic acid–water (A) and 0.1% formic acid–acetonitrile (B) at 0.3 mL/min flow rate, and the injection volume was 1 μL by an automatic sampling system. The elution gradient was set as follows: 0–2 min, 95% (A)-5% (B); 5–42 min, 95% (A)-5% (B) to 5% (A)-95% (B); 42–47 min, 5% (A)-95% (B); 47–47.1 min, 5% (A)-95% (B) to 95% (A)-5% (B); and 47.1–50 min, 95% (A)-5% (B). The instrumental settings of the Q-Orbitrap mass spectrometer were as follows: the spray voltage of the positive mode and the negative mode were, respectively, set at +3.0 kV and −2.5 kV, the probe heater temperature was 350 °C, the sheath gas was 35 arb, the auxiliary gas was 10 arb, the capillary temperature was 320 °C, and S-Lens RF level was 60. The primary resolution of the full scan was 70,000, with a scanning range from *m*/*z* 100 to 1500; the secondary resolution was 17,500, with a collision energy gradient of 20 ev, 40 ev and 60 ev. The data were acquired and processed by Compound Discover 2.1 and Xcalibur 4.0 software. The chemical constituents of *D. nobile* were identified based on quasi-molecular ion peak, fragment ion information, chromatographic retention time, and natural product databases.

### 3.4. Hypoglycemic Effect of D. nobile

Sprague-Dawley (SD) male rats (190 ± 10 g) were provided by Liaoning Changsheng Biotechnology Co., Ltd. (Shenyang, China) (SCXK-Liao 2020-0001). All rats were raised in standard cages under controlled temperature (25 ± 2 °C) and humidity (55 ± 10%) with a 12 h light/12 h dark cycle and with water and respective diet available *ad libitum*. The experiments were conducted in accordance with the principles of the Care and Use of Laboratory Animals, previously approved by the Animal Ethics Committee of Guizhou Medical University. After one week of acclimatization, all 40 rats were divided randomly into five groups: normal control group (NC), type 2 diabetes model group (DM), metformin (100 mg/kg/day) positive control group (PC), *D. nobile* extract (400 mg/kg/day) group (DNE), and *D. nobile* polysaccharide (400 mg/kg/day) group (DNP). The type 2 diabetic rat models were established by a high-fat diet and STZ intraperitoneal injection for evaluating hypoglycemic activity and mechanisms of *D. nobile*; all rats, except those in the normal control group, were fed with a high-fat diet including 59% basic diet, 20% sucrose, 18% lard oil, and 3% egg during the experiment, while the rats in the normal control group were given a basic diet. After 8 weeks of dietary intervention, a dose of 35 mg/kg STZ (dissolved with 0.1 mol/L citrate buffer, pH 4.2) was given to the rats to be used as the diabetic disease models, and the normal rats were given the same dose of citrate buffer. Blood samples were collected from the rats’ tail veins, and the fasting blood glucose (FBG) levels were measured by a glucose meter after one week of injecting STZ; rats with FBG exceeding 16.7 mmol/L, polydipsia, polyuria and other symptoms were identified as successful type 2 diabetes models. The rats in each group were treated with the respective drugs by oral gavage continuously for 8 weeks, and the NC and DM groups were given 0.5%CMC-Na solution daily. During the drug treatment, FBG and body weight were measured weekly, and food intake and water intake were recorded at 0 and 8th week, respectively. An oral glucose tolerance test (OGTT) was performed at 8th week; all rats were orally administered glucose at a dose of 2 g/kg after 12 h of fasting, blood glucose level was measured from the tip of the tail at 0, 0.5, 1, 1.5 and 2 h, and the area under the curve (AUC) was calculated using the following formula: $\mathrm{AUC}=1/2\overset{n}{\underset{i=1}{\sum }}\mathrm{Xi}-1\left(\mathrm{Yi}-1+\mathrm{Yi}\right)$.

At the end of the animal experiment, all rats were fasted for 12 h, anaesthetized by an intraperitoneal injection of 20% urethane solution for rat blood collection from the abdominal aorta, sampled for biological tissue sampling, and then euthanized. The blood collected in blood collection tubes was placed at room temperature for 30 min, and then centrifuged at 4 °C and 3000 rpm for 15 min; the supernatant was taken, sub packaged and stored at −80 °C for biochemical index detection. Total cholesterol (TC), triacylglycerols (TG), high-density lipoprotein cholesterol (HDL-C), and low-density lipoprotein cholesterol (LDL-C) in the rat serum were determined by biochemical kits (Jiancheng Bioengineering, Nanjing, China). Liver glycogen (Solarbio, Beijing, China) and fasting serum insulin (FINS) (Elabscience Biotechnology, Wuhan, China) were also detected. The homeostatic model assessment of insulin resistance (HOMA-IR) was calculated by HOMA-IR = FBG (mmol/L) × FINS (mU/mL)/22.5. The liver, heart, spleen, lung, kidney and pancreas were also collected, measured for organ coefficients, sub packaged and stored at −80 °C. Some liver and pancreas were fixed in 4% paraformaldehyde for histopathological analysis. When the liver and pancreas tissue samples were further fixed in 10% neutral buffered formalin, embedded in paraffin, trimmed longitudinally and routinely processed, these tissue samples were then cut into 5–7 μm thick section on a rotary microtome and mounted on a APES-coated glass slide. Finally, the tissue section was stained with hematoxylin and eosin (H&E) and analyzed.

### 3.5. Hypoglycemic Mechanism of D. nobile

#### 3.5.1. Network Pharmacological Analysis

All above-identified chemical compounds in the DNE through UPLC-ESI-Q-Obitrap MS analysis were evaluated for their pharmaceutical properties (GIA, gastrointestinal absorption and DL, druglikeness) by using the SwissADME database; the compounds would be regarded as medicinal compounds only when the predicted ADME results both met the requirements of “high” in GIA and ≥2 of 5 (Lipinski, Ghose, Veber, Egan and Muegge) conditions in DL [35,36]. The qualified compounds were imported into the SwissTargetPrediction and PharmMapper database, with the species being restricted to “Homo sapiens” for target prediction. GSE153315 as clinical expression profiling data including 10 T2DM patients and 10 controls was from the GEO database based on the microarray platform GPL17303. For further data processing, the probe ID was converted to a gene symbol, differentially expressed genes (DEGs) between the T2DM patients and healthy individuals were screened using R 4.1.3 software according to *p* < 0.05, and the|log_2_ fold change (FC)| > 1.5 were visualized by volcano plots and heatmap clusters. Putative T2DM genes were retained from the GeneCards database, with “type 2 diabetes mellitus” as keywords and screening genes with relevance score ≥ 10. The protein–protein interaction (PPI) was explored using the STRING database based on the overlapping targets among the candidate compound targets, GSE153315 targets and T2DM-related targets; the score of protein interaction was set as “medium confidence ≥ 0.4” to ensure data integrity. The Cytoscape 3.7.2 software was utilized to visualize “Herb-Compounds-Targets-Pathway-Disease” network and PPI network to screen for key hypoglycemic compounds in *D. nobile* and core targets, and the KEGG pathway enrichment analysis was also accomplished (*p* < 0.05) through the DAVID database.

#### 3.5.2. Molecular Docking Analysis

Based on the results of the bioinformatic network analysis, four core targets, including AKT1 (PDB ID: 4EJN), PIK3CA (PDB ID: 4JPS), GSK3B (PDB ID: 4ACH) and EGFR (PDB ID: 1XKK), were finally selected for molecular docking with ten key active compounds in *D. nobile*. The 3D structure of the target proteins was from the PDB database, and the solvent molecules and original ligands in the target proteins were cleaned up for the naked proteins, which were further hydrogenated, checked for charge and exported as the pdbqt files of the target proteins. The active compounds were also prepared as pdbqt files after energy minimization. Each docking pocket or site was defined from the active site of the original ligand, molecular docking was carried out by Autodock vina 1.1.2, and the optimal conformation of the docked models was analyzed and visualized by Proteins Plus Server and PyMOL 2.4.

#### 3.5.3. Western Blot

A total of 50 mg of liver tissue was homogenized in RIPA lysate for 5 min, and then centrifuged at 13,000 rpm at 4 °C for 10 min; the protein content was measured by using a BCA protein assay. Equal amounts of the protein samples were separated by 10% sodium dodecyl sulphate-polyacrylamide gel electrophoresis (SDS-PAGE) and electro-transferred to PVDF membrane. Subsequently, the membranes were blocked in 5% skim milk for 2 h at room temperature, and then incubated overnight with the primary antibodies against PI3K (1:1000), p-PI3K (1:500), AKT (1:1000), p-AKT (1:1000), GSK3β (1:1000), p-GSK3β (1:1000), GLUT4 (1:1000), PEPCK (1:5000), G6Pase (1:1000) and GAPDH (1:5000) on a shaker in a 4 °C refrigerator. After incubation with the secondary antibody for 1 h, the protein bands were visualized through the ECL luminescent solution, and the density values of the protein bands were finally quantified by the ImageJ 1.51 software.

### 3.6. Statistical Analysis

Statistical analysis was performed using GraphPad Prism 7.0, and the values were shown as mean ± SD. One-way analysis of variance (ANOVA) followed by Duncan’s post hoc test was used to compare the group differences, and statistical significance was set at *p* < 0.05.

## 4. Conclusions

An UPLC-ESI-Q-Orbitrap, network pharmacology and in vivo experimental verification was firstly established for a rapid identification of hypoglycemic compounds of *D. nobile* in this study. A total of 39 potential hypoglycemic compounds were characterized. α-Linolenic acid, dihydroconiferyl dihydro-p-coumarate, naringenin, *trans-N*-feruloyltyramine, gigantol, moscatilin, 4-*O*-methylpinosylvic acid, venlafaxine, nordendrobin and tristin were considered as the key hypoglycemic compounds of *D. nobile*. The hypoglycemic mechanism of *D. nobile* may be related to the activation of the insulin signaling pathway, thus improving this disorder of glucose and lipid metabolism and insulin resistance. These findings provide us a clearer material basis for *D. nobile*, which can be used to guide new hypoglycemic drug development using *D. nobile*.

## Figures and Tables

**Table 1:** Identified chemical constituents in *D. nobile* by UPLC-ESI-Q-Orbitrap.

No.	t_R_ (min)	Ion	MS^1^ (*m*/*z*)	Formula	Diff (ppm)	MS^2^ (*m*/*z*)	Identification	Category	CAS	InChIKey
DN1	0.66	[M-H]^−^	341.10809	C_12_H_22_O_11_	0.739	179.05489, 161.04433, 143.03384, 89.02295, 71.01239, 59.01244	Sucrose	Glycoside	57-50-1	CZMRCDWAGMRECN-UGDNZRGBSA-N
	0.68	[M+Na]^+^	365.10434		−2.992	203.05199, 185.04161				
DN2	0.71	[M-H]^−^	191.05507	C_7_H_12_O_6_	0.29	173.04453, 155.03311, 137.02310, 127.03867, 109.02816, 93.03314	Quinic acid	Other	77-95-2	AAWZDTNXLSGCEK-LNVDRNJUSA-N
DN3	0.85	[M+H]^+^	144.10167	C_7_H_13_NO_2_	1.632	98.09663, 84.08115, 70.06570, 58.06583	Homocycloleucine	Alkaloids	2756-85-6	WOXWUZCRWJWTRT-UHFFFAOYSA-N
DN4	0.96	[M+H]^+^	130.04958	C_5_H_7_NO_3_	2.227	102.0553, 84.04489, 70.06570, 56.05017	*L*-Pyroglutamic acid	Alkaloids	98-79-3	ODHCTXKNWHHXJC-VKHMYHEASA-N
DN5	1.07	[M+H]^+^	268.10291	C_10_H_13_N_5_O_4_	2.836	136.06163, 119.03513, 85.02884	Adenosine	Glycoside	58-61-7	OIRDTQYFTABQOQ-KQYNXXCUSA-N
DN6	3.08	[M+H]^+^	280.18994	C_16_H_25_NO_3_	2.784	262.18994, 234.18460, 220.13251, 166.12218	6-hydroxydendrobine	Alkaloids	7668-75-9	QSNCUGULHPBRGR-FYPZSMAZSA-N
DN7	3.57	[M+H]^+^	158.08104	C_7_H_11_NO_3_	0.821	112.07591, 84.04493, 70.06568, 56.05017	*N*-acetyl-*D*-proline	Alkaloids	59785-68-1	GNMSLDIYJOSUSW-ZCFIWIBFSA-N
DN8	6.06	[M-H]^−^	121.02806	C_7_H_6_O_2_	−2.445	93.03320, 65.03816	p-Hydroxybenzaldehyde	Other	123-08-0	RGHHSNMVTDWUBI-UHFFFAOYSA-N
DN9	8.99	[M+H]^+^	278.21063	C_17_H_27_NO_2_	−2.968	105.06984, 58.06584	Venlafaxine	Alkaloids	93413-69-5	PNVNVHUZROJLTJ-UHFFFAOYSA-N
DN10	9.68	[M+H]^+^	264.19507	C_16_H_25_NO_2_	−2.784	246.18503, 236.20000, 218.18958, 176.14381, 145.10034	Dendrobine	Alkaloids	2115-91-5	RYAHJFGVOCZDEI-UFFNCVEVSA-N
DN11	9.96	[M+H]^+^	250.17961	C_15_H_23_NO_2_	−2.181	232.16867, 204.17422, 145.10074, 133.10080, 105.07002	Nordendrobin	Alkaloids	73806-55-0	VUMQHLSPUAFKKK-UHFFFAOYSA-N
DN12	10.32	[M+H]^+^	285.16846	C_15_H_24_O_5_	−4.174	267.15814, 249.14789, 145.10077, 133.10095, 119.08533, 105.06994	Dendronobilin B	Other	-	ROURONGLEFVLGL-RQJMXFNBSA-N
	10.73	[M+Na]^+^	307.15051		−3.533	289.13968, 157.06676				
DN13	10.37	[M-H]^−^	593.14978	C_27_H_30_O_15_	−0.534	503.11746, 473.10797, 383.07654, 353.06586, 325.07150, 297.07630, 93.03333	Vicenin II	Glycoside	23666-13-9	FIAAVMJLAGNUKW-VQVVXJKKSA-N
DN14	10.91	[M+H]^+^	267.15805	C_15_H_22_O_4_	−3.877	249.14771, 231.13695, 203.14226, 155.06976, 96.08576	Verrucarol	Other	2198-92-7	ZSRVBNXAPSQDFY-OJVARPOJSA-N
DN15	11.17	[M+H]^+^	235.16855	C_15_H_22_O_2_	−3.004	217.15810, 205.15822, 189.16325, 175.11128, 161.09561, 147.11646, 133.10085	Curcumenol	Other	19431-84-6	ISFMXVMWEWLJGJ-NZBPQXDJSA-N
DN16	11.24	[M+H]^+^	306.20541	C_18_H_27_NO_3_	−3.136	249.14839, 175.14801, 121.10115	Unknown 1	-	-	-
DN17	13	[M+H]^+^	183.07768	C_6_H_15_O_4_P	−2.141	155.04639, 127.01531, 98.98456	Triethyl phosphate	Other	78-40-0	DQWPFSLDHJDLRL-UHFFFAOYSA-N
DN18	13.83	[M-H]^−^	461.23892	C_22_H_38_O_10_	1.726	179.05470, 161.04404, 119.03348	2-(4-Methyl-3-cyclohexen-1-yl)-2-propanyl-6-O-(6-deoxy-α-L-mannopyranosyl)-β-D-glucopyranoside	Glycoside	-	-
DN19	15.05	[M+H]^+^	219.17361	C_15_H_22_O	−3.339	163.11115, 145.10080, 131.08527, 105.07000	α-Cyperone or Germacrone	Other	4674-50-4	WTOYNNBCKUYIKC-JMSVASOKSA-N
DN20	15.22	[M-H]^−^	312.12369	C_18_H_19_NO_4_	2.1	297.10040, 190.04984, 178.04990, 148.05173, 135.04387	*Trans-N*-feruloyltyramine	Alkaloids	66648-43-9	NPNNKDMSXVRADT-WEVVVXLNSA-N
DN21	16.28	[M+H]^+^	219.17357	C_15_H_22_O	−3.522	159.11636, 145.10075, 107.08556, 105.06995	Germacrone or α-Cyperone	Other	6902-91-6	CAULGCQHVOVVRN-SWZPTJTJSA-N
DN22	16.42	[M-H]^−^	259.09708	C_15_H_16_O_4_	2.295	244.07341, 137.02312, 121.02820	Tristin	Bibenzyl	139101-67-0	KPFFMALTIRFAHW-UHFFFAOYSA-N
DN23	17.09	[M-H]^−^	271.06073	C_15_H_12_O_5_	2.324	119.04885, 151.00240, 107.01246	Naringenin	Flavonoids	67604-48-2	FTVWIRXFELQLPI-UHFFFAOYSA-N
DN24	18.69	[M+H]^+^	305.1369	C_17_H_20_O_5_	4.753	181.08534, 166.06190, 151.07495, 137.05937, 119.04906, 91.05453	Moscatilin	Bibenzyl	108853-14-1	YTRAYUIKLRABOQ-UHFFFAOYSA-N
DN25	18.99	[M-H]^−^	329.23279	C_18_H_34_O_5_	1.638	229.14378, 211.13306, 171.10156, 139.11140	(15Z)-9,12,13-Trihydroxy-15-octadecenoic acid	Other	-	DNWUYCUUEGGVPR-CLTKARDFSA-N
DN26	19.11	[M+H]^+^	245.11673	C_15_H_16_O_3_	−2.003	227.10631, 213.09059, 151.07495, 137.05936, 121.06474, 103.05436, 93.07018	Batatasin III	Bibenzyl	56684-87-8	VYQXIUVIYICVCM-UHFFFAOYSA-N
	19.14	[M-H]^−^	243.10201		1.806	227.07059, 213.05479, 151.03877, 93.03316				
DN27	20.71	[M+H]^+^	228.26805	C_15_H_33_N	−2.307	85.10143, 71.08615, 60.08149	Dodecyltrimethylammonium	Other	10182-91-9	VICYBMUVWHJEFT-UHFFFAOYSA-N
DN28	20.77	[M-H]^−^	329.1387	C_19_H_22_O_5_	1.063	208.07326, 181.08633, 179.07018, 149.05962, 121.02794	Dihydroconiferyl dihydro-p-coumarate	Other	-	ZXQHQUXJMNGVJP-UHFFFAOYSA-N
DN29	20.91	[M+H^]+^	275.12692	C_16_H_18_O_4_	−3.146	243.10115, 151.07492, 137.05934, 121.06466, 107.04920	Gigantol	Bibenzyl	67884-30-4	SDXKZPQOVUDXIY-UHFFFAOYSA-N
	20.84	[M-H]^−^	273.1127		−2.819	258.08911, 137.02310, 121.02808, 109.02803				
DN30	21.05	[M+H]^+^	273.11145	C_16_H_16_O_4_	−2.510	255.10074, 241.08511, 213.09032, 195.07991, 182.07214, 170.07227	Ephemeranthol B	Phenanthrene	130827-44-0	SOHTUOALNFQEMJ-UHFFFAOYSA-N
	21.21	[M+H]^−^	271.09711		2.304	256.07352, 241.04985, 224.04697, 195.04405, 185.05943, 167.04875				
DN31	21.4	[M+H]^+^	271.09546	C_16_H_14_O_4_	3.783	253.08495, 238.06938, 225.09015, 210.06674, 182.07204, 165.06932	Nudol or 4-*O*-Methylpinosylvic acid	Phenanthrene or Bibenzyl	86630-46-8	JZIYNZGPIKGKQC-UHFFFAOYSA-N
DN32	22.37	[M-H]^−^	241.08632	C_15_H_14_O_3_	1.656	241.08617, 226.06265, 198.06729, 182.07178, 169.06473	2-Methoxy-9,10-dihydrophenanthrene-4,5-diol	Phenanthrene	-	KQMGXHNRKZYDEK-UHFFFAOYSA-N
DN33	28.17	[M+H]^+^	279.23123	C_18_H_30_O_2_	2.244	261.22159, 209.15300, 195.13731, 123.11677, 109.10130, 95.08582	α-Linolenic acid	Other	463-40-1	DTOSIQBPPRVQHS-PDBXOOCHSA-N
DN34	28.51	[M+H]^+^	271.09512	C_16_H_14_O_4_	−4.869	253.08514, 238.06178, 225.09023, 210.06693, 182.07220, 165.06952	4-*O*-Methylpinosylvic acid or Nudol	Bibenzyl or Phenanthrene	149697-30-3	PICDNGANOHNCPT-BQYQJAHWSA-N
DN35	30.15	[M-H]^−^	303.15955	C_18_H_24_O_4_	1.532	259.17096, 205.04944, 161.05963	Unknown 2	-	-	-
DN36	34.17	[M+H]^+^	256.26239	C_16_H_33_NO	−4.297	144.13774, 130.12199, 116.10680, 102.09145, 88.07596, 74.06048	Hexadecanamide	Other	629-54-9	HSEMFIZWXHQJAE-UHFFFAOYSA-N
DN37	35.68	[M-H]^−^	339.23215	C_23_H_32_O_2_	0.865	163.1116, 147.08009, 107.04906	2,2′-Methylenebis(4-methyl-6-tert-butylphenol)	Other	119-47-1	KGRVJHAUYBGFFP-UHFFFAOYSA-N
DN38	36.07	[M-H]^−^	279.23236	C_18_H_32_O_2_	1.802	175.20976	Unknown 3	-	-	-
DN39	37.89	[M+H]^+^	284.29401	C_18_H_37_NO	−2.748	186.18478, 144.13776, 130.12242, 116.10702, 102.09155, 88.07607	Stearamide	Other	124-26-5	LYRFLYHAGKPMFH-UHFFFAOYSA-N
DN40	40.18	[M+H]^+^	165.0907	C_10_H_12_O_2_	1.855	147.08064, 137.09578, 119.08543, 109.10125, 91.05452	4-Phenylbutyric acid	Other	1821-12-1	OBKXEAXTFZPCHS-UHFFFAOYSA-N
DN41	41.29	[M+H]^+^	338.34058	C_22_H_43_NO	−3.433	321.31415, 226.21616, 149.13222, 135.11647	Erucamide	Other	112-84-5	UAUDZVJPLUQNMU-KTKRTIGZSA-N
DN42	48.82	[M+H]^+^	141.11308	C_16_H_12_N_4_	−2.005	112.08706, 98.07157, 85.07648, 71.06096	Hexamethylenetetramine	Alkaloids	100-97-0	VKYKSIONXSXAKP-UHFFFAOYSA-N

Note: DN: compound from *D. nobile*. MS^1^: parent ions. MS^2^: grade 1 fragment ions derived from the parent ion in the DDA mode.

**Table 2:** Effects of *D. nobile* on the organ coefficients of rats.

Items	NC	DM	DNP	DNE	PC
Heart coefficient (%)	0.28 ± 0.03	0.44 ± 0.082 ^##^	0.31 ± 0.07 *	0.34 ± 0.07	0.31 ± 0.04 *
Liver coefficient (%)	3.05 ± 0.17	5.38 ± 0.96 ^###^	3.87 ± 0.96 *	3.93 ± 0.75 *	3.69 ± 0.59 **
Spleen coefficient (%)	0.17 ± 0.02	0.22 ± 0.04 ^#^	0.17 ± 0.03 *	0.14 ± 0.02 ***	0.12 ± 0.01 ***
Lung coefficient (%)	0.53 ± 0.15	0.65 ± 0.08	0.66 ± 0.10	0.71 ± 0.16	0.55 ± 0.11
Kidney coefficient (%)	0.68 ± 0.07	1.09 ± 0.10 ^###^	0.86 ± 0.15 *	0.90 ± 0.14 *	0.80 ± 0.06 **
Pancreas coefficient (%)	0.56 ± 0.08	0.29 ± 0.02 ^###^	0.40 ± 0.02 *	0.43 ± 0.07 **	0.37 ± 0.09
Final body weight (g)	500.60 ± 41.02	323.30 ± 39.66 ^###^	362.20 ± 31.10 *	388.30 ± 11.18	355.00 ± 44.41

Note: Compared with the NC group, ^#^ *p* < 0.05, ^##^ *p* < 0.01, and ^###^ *p* < 0.001; compared with the DM group, * *p* < 0.05, ** *p* < 0.01, and *** *p* < 0.001.

**Table 3:** Effect of *D. nobile* on serum lipid profile, insulin level and liver glycogen of rats.

Items	NC	DM	DNP	DNE	PC
TC (mmol/L)	1.14 ± 0.08	2.65 ± 0.17 ^###^	1.22 ± 0.11 ***	1.21 ± 0.15 ***	1.45 ± 0.15 ***
TG (mmol/L)	0.34 ± 0.09	1.77 ± 0.11 ^###^	0.82 ± 0.12 ***	0.54 ± 0.14 ***	0.44 ± 0.16 ***
LDL-C (mmol/L)	0.68 ± 0.07	1.18 ± 0.08 ^###^	0.68 ± 0.06 ***	0.71 ± 0.07 ***	0.75 ± 0.07 ***
HDL-C (mmol/L)	1.15 ± 0.06	0.97 ± 0.19	0.93 ± 0.09	1.03 ± 0.18	1.15 ± 0.10
FINS (mIU/L)	12.55 ± 2.86	22.40 ± 2.61 ^###^	15.56 ± 3.36 **	14.36 ± 3.03 ***	14.33 ± 1.85 ***
HOMA-IR	2.31 ± 0.42	25.67 ± 3.73 ^###^	10.93 ± 3.92 ***	10.11 ± 1.54 ***	9.63 ± 2.89 ***
Liver glycogen (mg/g)	17.96 ± 3.14	5.92 ± 1.63 ^###^	15.56 ± 1.55 ***	9.47 ± 1.66	13.55 ± 3.35 **

Note: Compared with the NC group, ^###^ *p* < 0.001; compared with the DM group, ** *p* < 0.01 and *** *p* < 0.001.

## Data Availability

Data are contained within the article.

## Abbreviations

APES:	3-aminopropyl triethoxysilane
DDA:	data-dependent acquisition
DN:	compound from *Dendrobium nobile*
DNE:	*Dendrobium nobile* extract
DNP:	*Dendrobium nobile* polysaccharide
ESI:	electron spray ionization
HRMS:	high-resolution mass spectrometry
PPI:	protein–protein interaction
T2DM:	Type 2 diabetic mellitus
TIC:	total ions current
